# A Genetic Mechanism for Emergence of Races in *Fusarium oxysporum* f. sp. *lycopersici*: Inactivation of Avirulence Gene *AVR1* by Transposon Insertion

**DOI:** 10.1371/journal.pone.0044101

**Published:** 2012-08-27

**Authors:** Keigo Inami, Chizu Yoshioka-Akiyama, Yasuaki Morita, Mutsuko Yamasaki, Tohru Teraoka, Tsutomu Arie

**Affiliations:** 1 Graduate School of Agriculture, Tokyo University of Agriculture and Technology (TUAT), Fuchu, Japan; 2 Kochi Agricultural Research Center, Nangoku, Japan; Nanjing Agricultural University, China

## Abstract

Compatible/incompatible interactions between the tomato wilt fungus *Fusarium oxysporum* f. sp. *lycopersici* (*FOL*) and tomato *Solanum lycopersicum* are controlled by three avirulence genes (*AVR1*–*3*) in *FOL* and the corresponding resistance genes (*I*–*I3*) in tomato. The three known races (1, 2 and 3) of *FOL* carry *AVR* genes in different combinations. The current model to explain the proposed order of mutations in *AVR* genes is: i) *FOL* race 2 emerged from race 1 by losing the *AVR1* and thus avoiding host resistance mediated by *I* (the resistance gene corresponding to *AVR1*), and ii) race 3 emerged when race 2 sustained a point mutation in *AVR2,* allowing it to evade *I2*-mediated resistance of the host. Here, an alternative mechanism of mutation of *AVR* genes was determined by analyses of a race 3 isolate, KoChi-1, that we recovered from a Japanese tomato field in 2008. Although KoChi-1 is race 3, it has an *AVR1* gene that is truncated by the transposon *Hormin*, which belongs to the *hAT* family. This provides evidence that mobile genetic elements may be one of the driving forces underlying race evolution. KoChi-1 transformants carrying a wild type *AVR1* gene from race 1 lost pathogenicity to cultivars carrying *I*, showing that the truncated KoChi-1 *avr1* is not functional. These results imply that KoChi-1 is a new race 3 biotype and propose an additional path for emergence of *FOL* races: Race 2 emerged from race 1 by transposon-insertion into *AVR1*, not by deletion of the *AVR1* locus; then a point mutation in race 2 *AVR2* resulted in emergence of race 3.

## Introduction

In the arms race between plants and pathogens, the pathogens can win by circumventing the immune system of host plants, *e.g.,* by avoiding or suppressing defense mechanisms. In general, plants have two types of resistance: polygenic (horizontal), controlled by multiple genes, each with a small phenotypic effect, and monogenic (vertical), controlled by a single resistance (*R*) gene, which often confers a high level of resistance [1]. Monogenic resistance generates immune responses (*e.g*. hypersensitive reaction, HR) to particular pathogen(s) [1], and has been effective and practical to use in modern plant breeding. This resistance is described by the ‘gene-for-gene theory’ [2], which explains the relationship between pathogen races and host plant cultivars by the interaction between an avirulence (*AVR*) gene in the race and an *R* gene in the cultivar. When a race possessing an *AVR* gene attacks a cultivar carrying the corresponding *R* gene, resistance is induced in the plant and the disease does not occur. A loss of function in an *AVR* gene allows the pathogen to avoid induction of resistance in the cultivar, the pathogen gains pathogenicity to that cultivar, and a new pathogenic race has emerged.

The ascomycete *Fusarium oxysporum* Schlecht. emend. Snyd. et Hans. causes vascular diseases of many plant species, yet each strain of this fungus has strictly defined host specificity [3]. Strains that cause wilt disease only on tomato (*Solanum lycopersicum* L.) are classified as f. sp. *lycopersici* Snyd. et Hans. (*FOL*). Three races of *FOL* have been reported; their relationship with tomato cultivars is explained by the ‘gene-for-gene theory’ [4]. Original descriptions of *FOL* races 1, 2 and 3 appeared before 1895 in England, in 1939 in the USA and in 1978 in Australia, respectively [5]. In Japan, races 1, 2 and 3 were reported in Fukuoka in 1905, in 1966 and in 1997, respectively [6].

To date, the *R* genes *I*, *I2* and *I3* are known in tomato cultivars [7]; these *R* genes correspond to the avirulence genes *AVR1*, *AVR2* and *AVR3* in *FOL*, respectively (Table 1). Historically, race 1-resistant cultivars (*I i2 i3*), races 1 and 2-resistant cultivars (*I I2 i3*), and races 1, 2 and 3-resistant cultivars (*I I2 I3*) have been bred sequentially, each genotype corresponds to the emergence of a new race.

**Table 1:** Relationship between FOL races and tomato cultivars.

	Tomato cultivar (*R* gene^b^)			
*FOL* race *(AVR* gene^a^)	Ponderosa	Momotaro	Walter	Block
	(*i i2 i3*)	(*I i2 i3*)	(*I I2 i3*)	(*I I2 I3*)
1 (*AVR1 AVR2 AVR3*)	S	R	R	R
2 (*– AVR2 AVR3*)	S	S	R	R
3 (*– avr2 AVR3*)	S	S	S	R

S, compatible; R, incompatible.

a –, loss of the AVR1 locus; avr2, allele containing a point mutation in the ORF [9].

The *FOL AVR* genes (*AVR1, AVR2* and *AVR3*) are unique to *FOL* [8], [9], [10] and are carried in different combinations in different *FOL* races (Table 1). *AVR1* ( = *SIX4*) is unique to race 1 [11], whereas *AVR2* ( = *SIX3*) is found in races 1 and 2. Three nucleotide substitutions (G121A, G134A and G137C) in *AVR2*, which cause loss of avirulence function (*avr2*) have been found in race 3 [9]. *AVR3* ( = *SIX1*), which exists in all races [12], is known to have two silent mutations (lysine or glutamine at amino acid 164) that do not influence avirulence to *I3* cultivars [13]. *FOL* races can be determined by *AVR* gene combinations [11], [14].

Based on the knowledge of *AVR* genes, it was suggested that *FOL* races emerged as follows [9]: race 1 (*AVR1 AVR2 AVR3*) lost the *AVR1* locus and became race 2 (– *AVR2 AVR3*), which escapes recognition by the *I* gene; a nucleotide substitution in race 2 *AVR2* resulted in race 3 (– *avr2 AVR3*), which evades recognition by both *I* and *I2*. Those mutations of *AVR* genes are consistent in many *FOL* isolates [11].

Mating type (MAT), vegetative compatibility group (VCG), and phylogeny have been used to characterize genetic relationships among *FOL* isolates [15], [16], [17]. MAT and VCG correlate with the phylogenetic relationship [16]. All *FOL* isolates belong to one of three clades (A1–A3) in the *F. oxysporum* phylogeny based on the intergenic region of ribosomal DNA (rDNA-IGS), suggesting a polyphyletic relationship with at least three *FOL* origins [16], [17]. In Japanese isolates, race correlates with the phylogenetic relationship; races 1, 2 and 3 belong to clades A2, A1 and A3, respectively [16].

Masunaga el al. first reported emergence of race 3 in Japan in 1997 [18]. It is now the number one wilt disease problem in Japan, since most commercial tomato cultivars are resistant to races 1 and 2 but susceptible to race 3. Japanese race 3 isolates all group in clade A3 and are MAT1-2 and VCG 0033 [16].

In 2008, a new outbreak of Fusarium wilt caused devastating damage to tomato production in greenhouses in Hidaka, Kochi Prefecture, Japan (Fig. S1A, B). The genotype of the affected cultivar was *I I2 i3*, which suggested the presence of race 3. However, certain characteristics of the pathogenic isolate did not match those reported for previously described Japanese race 3 isolates, suggesting a different biotype, and tomato wilt caused by the novel biotype of *FOL* race 3 has been occurring in Kochi to date. In this study, the novel biotype was analyzed by phenotypic, genetic and phylogenetic criteria; results suggest a new path for emergence of races.

## Results and Discussion

### A race 3 isolate, KoChi-1, belongs to a different lineage from the known race 3 isolates in Japan

**Figure 1:**
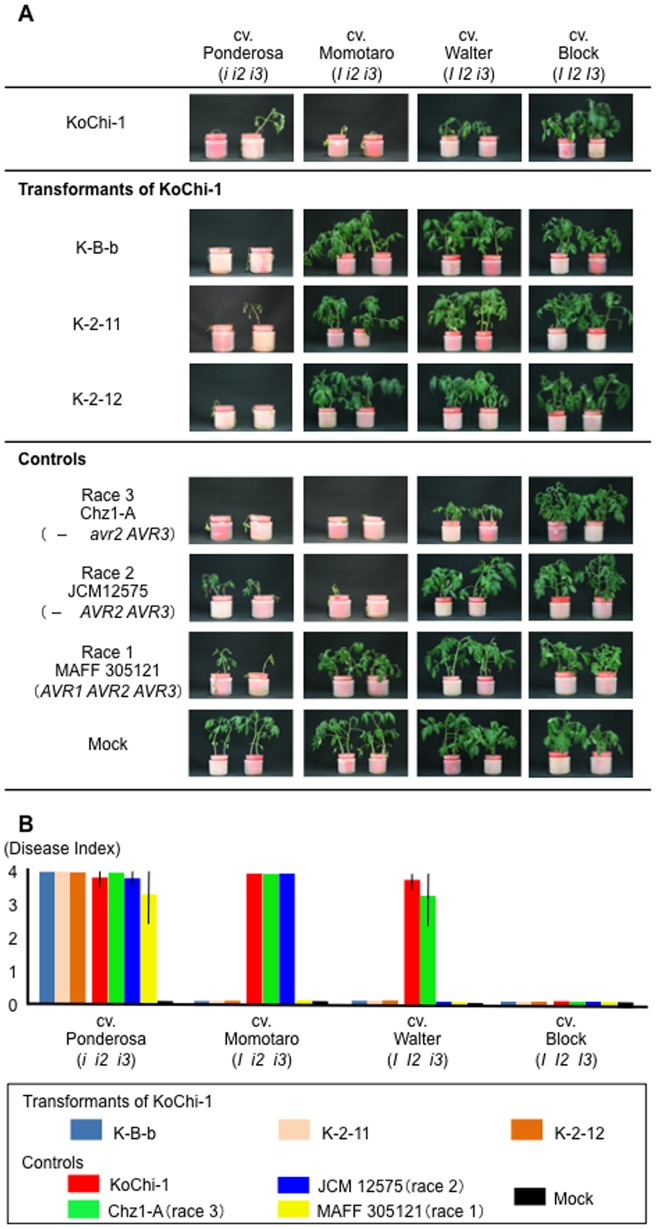
Virulence of KoChi-1 and its transformants. (A) KoChi-1 and its *AVR1*-complements were subjected to pathogenicity evaluation using four tomato cultivars, Ponderosa (*i i2 i3*), Momotaro (*I i2 i3*), Walter (*I I2 i3*) and Block (*I I2 I3*). The cv. Ponderosa does not have resistance to all *FOL* races, cv. Momotaro is resistant to *FOL* race 1 and susceptible to races 1 and 2, cv. Walter is susceptible to race 3 and resistant to races 1 and 2, and cv. Block is resistant to all *FOL* races. Inocula are as follows: KoChi-1 and its three transformants, K-B-b, K-2-11 and K-2-12; controls, race 1 MAFF 305121 (*AVR1 AVR2 AVR3*), race 2 JCM 12575 (– *AVR2 AVR3*) and race 3 Chz1-A (– *avr2 AVR3*). As a negative control, sterilized water was used (Mock). After three weeks of inoculation. (B) The disease severity of each individual was evaluated on external symptoms with 0∼4 scale, respectively. The external symptoms were scored as follows: 0, no wilt or yellowing; 1, lower leaves are yellowing; 2, lower and upper leaves are yellowing; 3, lower leaves are yellowing and wilt and upper leaves are yellowing; 4, all leaves are wilt and yellowing or dead. The symptoms were evaluated after three weeks of inoculation. Four plants were used in each isolate, with three replicates.

A fungal isolate from the vascular tissues of diseased tomato in a greenhouse in Kochi Prefecture, Japan was identified as *F. oxysporum* based on morphology [19] and nucleotide sequence of the rDNA-internal transcribed spacer (ITS) region (DDBJ/EMBL/GenBank accession No. AB675383). Characteristics of the isolate, designated KoChi-1, are summarized in Table 2. *In planta* assays showed that KoChi-1 caused wilt disease on cvs. Ponderosa (*i i2 i3*), Momotaro (*I i2 i3*) and Walter (*I I2 i3*), but not on cv. Block (*I I2 I3*), indicating that KoChi-1 was race 3 (Table 2; Fig. 1A, B). This result was consistent with the fact that the commercial cultivar grown in the greenhouse was Momotaro-Fight (*I I2 i3*, Takii Seed, Kyoto, Japan).

**Table 2:** Summary of characteristcis of KoChi-1 and other FOL isolates.

	Scores of wilt disease on tomato cultivar^a^									
*FOL* Isolate	Ponderosa	Momotaro	Walter	Block	*AVR1*	SNP in	Polymorphism	VCG	MAT	Phylogenetic
	(*i i2 i3*)	(*I i2 i3*)	(*I I2 i3*)	(*I I2 I3*)	locus^b^	*AVR2* c	in AVR3^d^			cladee
KoChi-1	3.75±0.25	4.0±0.0	3.75±0.25	0.0±0.0	*avr1*	G121A	E	0030+0032	1–1	A2
Race 3 (Chz1-A, Japan)	4.0±0.0	4.0±0.0	3.25±0.75	0.0±0.0	–	G121A	K	0033	1–2	A3
Race 3 (F240, USA)	nt	Nt	nt	nt	–	G134A	K	0030+0032	1–1	A2
Race 3 (NRRL 26383, USA)	nt	Nt	nt	nt	–	G121A	K	0033	1–2	A3
Race 2 (JCM 12575, Japan)	3.75±0.25	4.0±0.0	0.0±0.0	0.0±0.0	–	wt	K	0031	1–1	A1
Race 1 (MAFF 103036, Japan)	3.25±0.75	0.0±0.0	0.0±0.0	0.0±0.0	*AVR1*	wt	E	0030+0032	1-1	A2

a Four plants were used for each FOL isolate. The scores of external symptoms, using 0 (no symptoms) to 4 (death) scale are shown with standard error. All negative controls (inoculated with sterilized water) was 0.0±0.0 in all cultivars. These detailed results correspond to Fig. 2A, B. nt, not tested in this study.

b AVR1, carrying functional AVR1 gene; avr1, carrying AVR1 truncated by Hormin; –, null.

c wt, no SNPs; G121A indicates that 121st guanine was substituted to alanine.

d Mutation at the 164 amino acid of AVR3 (E = glutamine, K = lysine).

e Corresponds to Figure 3 and previous study [16].

Previous studies found that all race 3 isolates obtained in Japan (representative isolate Chz1-A is presented in Table 2) grouped in the A3 clade [16] (Table 2; Fig. S2), and were MAT1-2 and VCG 0033. However, we found that KoChi-1 belongs to the A2 clade (Table 2; Fig. S2), and is MAT1-1 and VCG 0030+0032. The A2 clade has been reported to include only race 1 isolates in Japan [16]. Taken together, these characteristics suggest KoChi-1 is a novel biotype of race 3, distinct from the race 3 isolates previously reported in Japan.

### KoChi-1 is the first reported race 3 isolate carrying the *AVR1* locus, which itself is truncated by a transposon

Although previously reported race 3 isolates (*e.g.*, Chz1-A) have no *AVR1* locus [8], [11], Southern blot analysis using an *AVR1* fragment from race 1 isolate MAFF 305121 (733 bp, nt 673–1406 bp, AB674509) as a probe presented that KoChi-1 possessed a single copy of *AVR1* in its genome (Fig. 2A).

**Figure 2:**
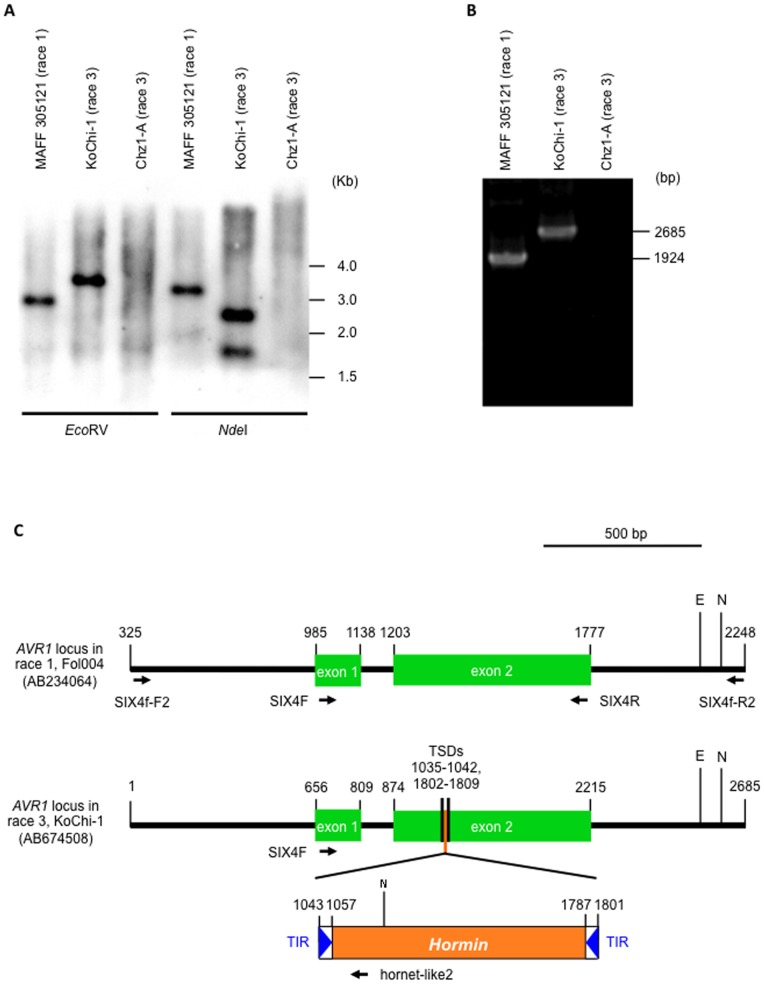
*AVR1* in KoChi-1 genome was truncated by a transposon *Hormin.* (A) Southern blot analysis to investigate the copy number of *AVR1* gene. *AVR1* probe was prepared using a primer set SIX4F/SIX4R (Table 3), and each gDNA was digested with restriction enzyme, *Eco*RV or *Nde*I (Fig. 2C). (B) Detection of *AVR1* locus from KoChi-1 using a primer set SIX4f-F2/SIX4f-R2 (Table 3, Fig. 2C). (C) Schematic representation of *AVR1* locus and *AVR1* gene truncated by a transposon *Hormin* (*avr1*). The nonautonomous transposon *Hormin* (759 bp, shown in orange square) is inserted in the second exon of *AVR1* in KoChi-1, *Hormin* harbors 15-bp tandem inverted repeats (TIRs, shown in blue triangle in white square) “CAGGGTTCAAATCCA” and 8-bp target site duplications (TSDs, shown with black line) “CACACCGG”. Arrows show primers. E, *Eco*RV site; N, *Nde*I site.

Then, we tried to amplify *AVR1* from KoChi-1 using a primer set SIX4f-F2/SIX4f-R2 designed by Rep & Houterman to amplify *AVR1* from race 1 (Table 3). The amplicon from KoChi-1 (2685 bp) was longer than that of MAFF 305121 (1924 bp) (Fig. 2B). The sequence of KoChi-1 *AVR1* was deposited in DDBJ/EMBL/GenBank databases with the accession No. AB674508. In this paper, nucleotide positions are assigned according to AB674508 unless otherwise stated.

**Table 3:** Primers used in this study.

Name	Sequence (5′-3′)	Targeting gene/Region	Reference
ITS1	TCCGTAGGTGAACCTGCGG	Ribsomal DNA internal transcribed spacer (ITS) region	[39]
ITS4	TCCTCCGCTT ATTGATATGC	Ribsomal DNA internal transcribed spacer (ITS) region	[39]
FIGS11	GTAAGCCGTCCTTCGCCTCG	Ribsomal DNA intergenic spacer (IGS) region	[16]
FIGS12	GCAAAATTCAATAGTATGGC	Ribsomal DNA intergenic spacer (IGS) region	[16]
SIX4F	ACTCGTTGTTATTGCTTCGG	*AVR1* (*SIX4*) gene	This study
SIX4R	CGGAGTGAAGAAGAAGCTAA	*AVR1* (*SIX4*) gene	This study
SIX3-F1	CCAGCCAGAAGGCCAGTTT	*AVR2* (*SIX3*) gene	[12]
SIX3-R2	GGCAATTAACCACTCTGCC	*AVR2* (*SIX3*) gene	[12]
FP962	TGAGCGGGCTGGCAATTC	*AVR2* (*SIX3*) gene	[46]
FP963	CAATCCTCTGAGATAGTAAG	*AVR2* (*SIX3*) gene	[46]
P12-F1	CCCCGAATTGAGGTGAAG	*AVR3* (*SIX1*) gene	[10]
P12-F2	GTATCCTCCGGATTTTGAGC	*AVR3* (*SIX1*) gene	[10]
P12-R1	AATAGAGCCTGCAAAGCATG	*AVR3* (*SIX1*) gene	[10]
SIX4f-F2	GTCGACTTAGAGTTTACTCC	*AVR1* locus (5′ flanking region)	Rep & Houterman (personal communication)
SIX4f-R2	ACTTAATTAATAGTCTGTTGTGT	*AVR1* locus (3′ flanking region)	Rep & Houterman (personal communication)
SIX4-in1	CCACTACCTTCTCCTTCCTT	*AVR1* locus (5′ flanking region)	This study
SIX4-in2	CTATCGCAGAGACGGGCATT	*AVR1* locus (exon 2)	This study
Gfmat1a	GTTCATCAAAGGGCAAGCG	*MAT1-1-1* alpha-box (*MAT1-1*)	This study
Gfmat1b	TAAGCGCCCTCTTAACGCCTTC	*MAT1-1-1* alpha-box (*MAT1-1*)	This study
GfHMG11	TACCGTAAGGAGCGTCAC	*MAT1-2-1* HMG-box (*MAT1-2*)	This study
GfHMG12	GTACTGTCGGCGATGTTC	*MAT1-2-1* HMG-box (*MAT1-2*)	This study
hornet-like2	CGTGGAATGGAATGGAATGG	Transposon *Hormin* in *avr1*	This study
FP157	ATGAAGTACACTCTCGCTACC	*FEM1*	[46]
FP158	GGTGAAAGTGAAAGAGTCACC	*FEM1*	[46]
Actin-f	AGGCACACAGGTGTTATGGT	*actin* (*S. lycopersicum*)	[47]
Actin-r	AGCAACTCGAAGCTCATTGT	*actin* (*S. lycopersicum*)	[47]

The structure of KoChi-1 *AVR1* was compared with that of the race 1 isolate Fol004 (nt 326–2248 in AM234064; Fig. 2C). KoChi-1 contained a different number (13 bp, nt 30–42) of contiguous guanines and one cytosine deletion (nt 2136, AM234064) in addition to a 759 bp-insertion. This small number of polymorphisms suggests that the *AVR1* locus is highly conserved. *AVR1* in race 1 is composed of two exons (154 and 575 bp) and one intron (64 bp), and encodes a protein of 242 amino acids [8] (Fig. S4), but the KoChi-1 *AVR1* sequence had a 759-bp insertion (nt 1043–1801) in exon 2.

BLASTN searches in the NCBI database suggested that the 759-bp insertion was a transposon with 15-bp terminal inverted repeats (TIRs; 5′-CAGGGTTCAAATCCA-3′; nt. 1043–1057, 1787–1801; Fig. 2C), and that both TIRs were flanked by 8-bp target site duplication (TSD; 5′-CACACCGG-3′; nt 1035–1042, 1802–1809; Fig. 2C). The sequence of the TIRs and the 5′ region of the transposon were highly homologous to the autonomous transposon *Hornet1* from *F. oxysporum* (AF076626) [20]. These characteristics are consistent with those of the *hAT* family of class II DNA transposons [21]. Hence, we have designated this transposon *Hormin* (*Hornet1* in miniature). *Hormin* does not encode transposases (and is therefore not autonomous) and may have emerged from *Hornet1* through a series of mutations. A transposon identical to *Hormin* was previously reported in the alcohol dehydrogenase gene *Adh1* in *FOL* NRRL 34936 [22]. This is the first report of an *F. oxysporum AVR* gene truncated by a transposon.

According to the Broad Institute *Fusarium* genome database website (http://www.broadinstitute.org/annotation/genome/fusarium_group/MultiHome.html), only 2 isolates, *FOL* race 2 NRRL 34936 (Spain, MAT1-1, VCG 0030) and *FOL* race 3 NRRL 54003 (USA, MAT1-2, VCG 0033), carried *Hormin*-identical sequences (72 and 2 copies, respectively) among 13 isolates of *Fusarium* spp. In NRRL 34936, *Hormin* was distributed on almost every chromosome (Fig. S3). On the other hand, *F. oxysporum* f. spp. *raphani* (NRRL 54004, pathogenic to radish and *Arabidopsis*), *pisi* (NRRL 37622, pathogenic to pea), *vasinfectum* (NRRL 25433, pathogenic to cotton), *melonis* (NRRL 26406, pathogenic to melon), *conglutinans* (PHW808, pathogenic to cabbage), and two *F. oxysporum* isolates (Fo47, a nonpathogenic isolate; FOSC 3-a, pathogenic to immunocompromised humans) had several *Hormin*-like (85.8∼99.8% homology) sequences.

### KoChi-1 *avr1* encodes a defective protein

**Figure 3:**
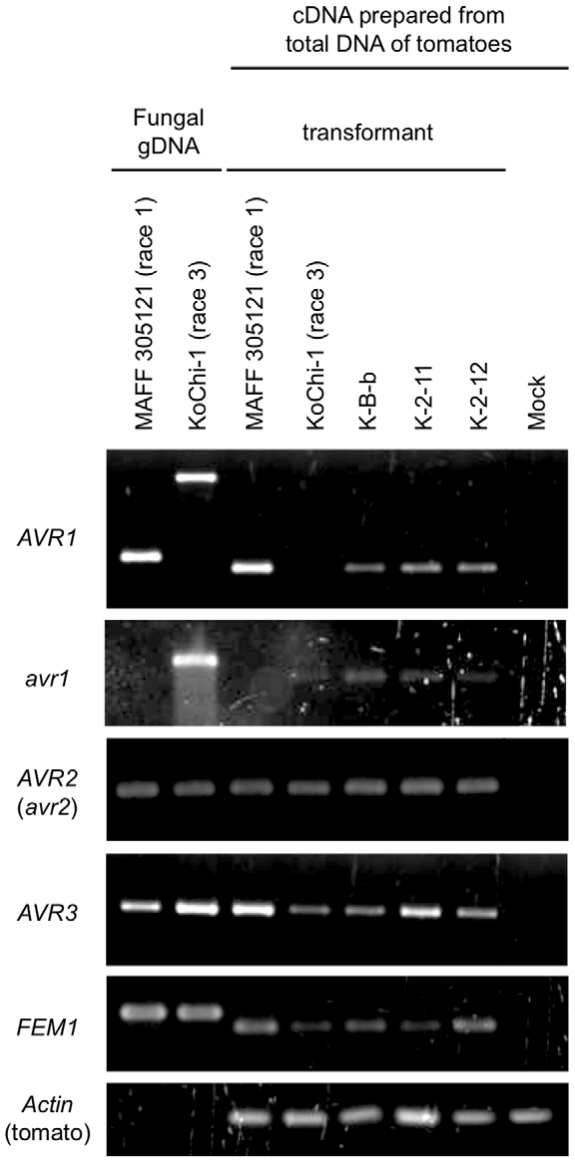
Gene expression of *AVR1*, *avr1*, *AVR2* (*avr2*) and *AVR3.* Eight days after inoculation with race 1 MAFF 305121 (*AVR1 AVR2 AVR3*), race 3 KoChi-1 (*avr1 avr2 AVR3*) and the three transformants (*avr1 AVR1 avr2 AVR3*); K-B-b, K-2-11 and K-2-12, total RNA was extracted from the roots of tomato (cv. Ponderosa) and investigated the transcription of genes *AVR1*, *avr1*, *AVR2* (*avr2*), *AVR3*, *FEM1* and *Actin* with the primer sets SIX4F/SIX4R, SIX4F/hornet-like2, FP962/FP963, P12-F1/P12-R1, FP157/FP158 and Actin-f/Actin-r, respectively (Table 3). *FEM1* and actin are used as controls for constitutively-expressed genes in fungal and plant tissues, respectively. Sterilized water is used as a negative control.

The deduced amino acid sequence of KoChi-1 *AVR1* with *Hormin* revealed a chimeric protein of 175 amino acids (avr1; Fig. S4) that may be nonfunctional. Here, we designate the *AVR1* gene truncated with *Hormin* as *avr1*. To investigate the transcription of *avr1*, total RNA was extracted from tomato roots inoculated with KoChi-1 or MAFF 305121 (race 1, as a control). RT-PCR using primer set SIX4F/SIX4R (designed to amplify *AVR1* including its intron) amplified a 734-bp fragment from MAFF 305121 RNA but not from KoChi-1 (Fig. 3). On the other hand, RT-PCR using primer SIX4F with primer hornet-like2 (designed on *Hormin*, see Table 3, Fig. 2C) generated a 440-bp fragment from KoChi-1 inoculated tomato only (Fig. 3), indicating that KoChi-1 *avr1* is expressed *in planta*. Neither *avr1* in KoChi-1 nor *AVR1* in MAFF 305121 was expressed in mycelia grown on PDB or MM medium (data not shown). This expression pattern was consistent with that of *AVR3* in *FOL* race 2 Fol007 [23].

### Other KoChi-1 *AVR* genes

KoChi-1 *avr2* contains the previously known point mutation G121A; it is one of three mutations known to cause loss of *AVR2* function in race 3 isolates [9]. KoChi-1 *AVR3* has a glutamine (E) type mutation (Table 2). To date, there have been no reports of E type *AVR3* mutations in race 3 [13]. Both *avr2* and *AVR3* of KoChi-1 were expressed during infection of tomato roots (Fig. 3).

### Complementation of KoChi-1 *avr1* with *AVR1* results in loss of pathogenicity to cultivars carrying the *I* gene

KoChi-1 (*avr1 avr2 AVR3*) was transformed with the Fol004 (race 1) *AVR1* gene. Each of three transformants (K-B-b, K-2-11 and K-2-12) had one copy of *AVR1* integrated ectopically into chromosomal DNA to yield strains with the genotype (*avr1 AVR1 avr2 AVR3*) (Fig. S5); the *AVR1* transgene was expressed (Fig. 3). Each of the three transformants lost pathogenicity to tomato cultivars carrying the *I* gene, *e.g.,* Momotaro (*I i2 i3*) and Walter (*I I2 i3*) (Fig. 1A, B). This confirms that *avr1* is not functional, and indicates that the mutation can be complemented by *AVR1*. It also indicates that the integrated *AVR1* functioned in spite of coexisting with *avr1*.

### How and where did KoChi-1 emerge?

**Figure 4:**
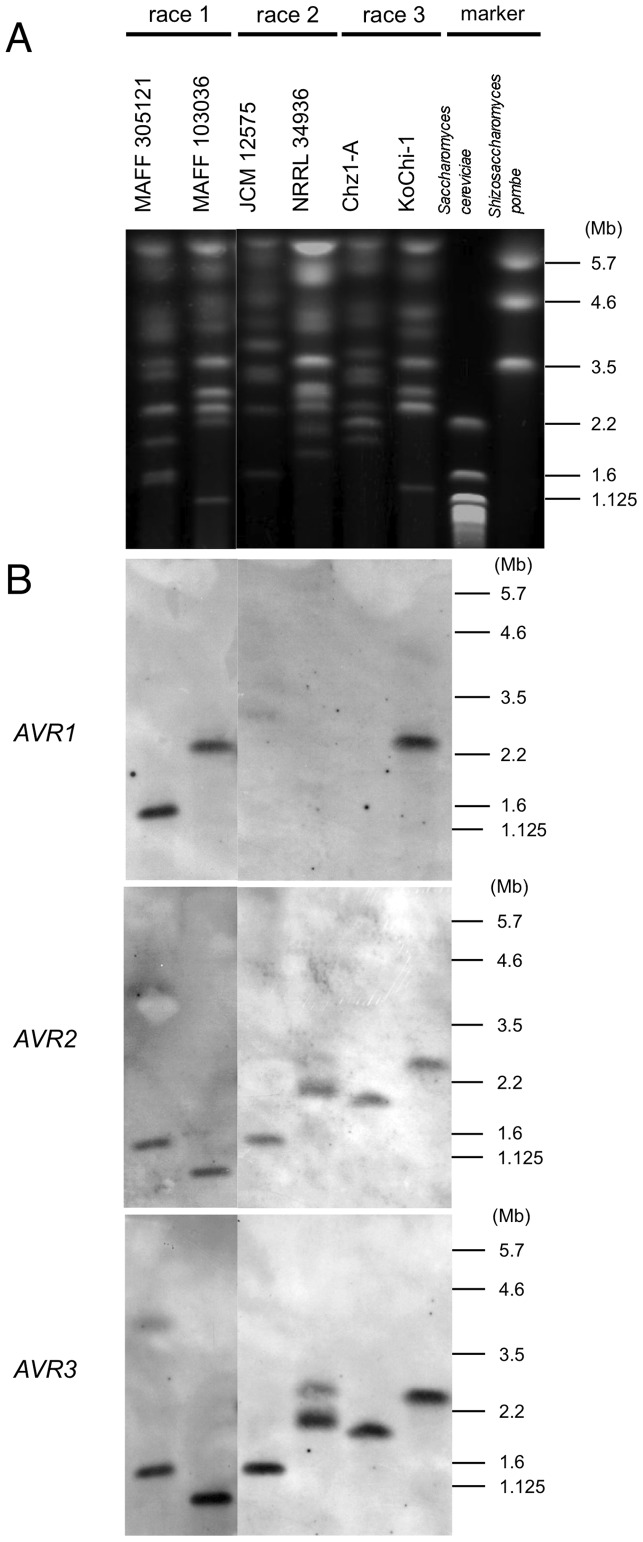
Localization of *avr1/AVR1, AVR2 and AVR3* on the chromosomes of KoChi-1 and other *FOL* isolates. (A) Karyotype of *FOL* isolates by CHEF-gel electrophoresis. Electrophoresis was performed in 1.0% Sea Kem gold agarose gel with CHEF Mapper XA Pulsed Field Electrophoresis System, as following condition; 260 hours run at 8°C, 1200-4800 s switch time at 1.5 V/cm. MAFF 305121 (*AVR1 AVR2 AVR3*, Japan); MAFF 103036 (*AVR1 AVR2 AVR3,* Japan); 73 (*AVR1 AVR2 AVR3*, Italy); Ita3 (*AVR1 AVR2 AVR3*, Italy); JCM 12575 (– *AVR2 AVR3*, Japan); NRRL 34936 (– *AVR2 AVR3*, Spain); Chz1-A (– *avr2 AVR3*, Japan); KoChi-1 (*avr1 avr2 AVR3*, Japan). The chromosomes of *Saccharomyces cerevisiae* and *Schizosaccharomyces pombe* were used as CHEF DNA size markers. (B) Southern blot analysis probed with *AVR1* (upper), *AVR2* (middle) and *AVR3* (bottom). Probes to detect *AVR1* (*avr1*), *AVR2* (*avr2*) and *AVR3* were prepared using primer sets SIX4F/SIX4R, SIX3-F1/SIX3R2 and P12-F2/P12-R1, respectively (Table 3).

According to the Broad Institute *Fusarium* genome database, *FOL* race 2 isolate NRRL 34936 bears *AVR2*, *AVR3* and genes encoding small proteins secreted into tomato xylem on a small (ca. 2.2 Mb) chromosome. Since the chromosomal location of *AVR1* is unknown, we investigated the location of KoChi-1 *avr1* by CHEF Southern hybridization (Fig. 4A, B). *avr1* was found on a small (ca. 2.5 Mb) chromosome together with *avr2* and *AVR3* (Fig. 4A, B; lane 8), which was also the case for *AVR1* in race 1 isolates MAFF 305121 (1.6 Mb; Fig. 4A, B; lane 1). The small chromosome of each isolate had different size. However, although MAFF 103036 (a Japanese race 1 isolate) was found to carry *AVR1* on a ca. 2.5 Mb chromosome, its *AVR2* and *AVR3* genes were found on a ca. 1.0 Mb chromosome (Fig. 4A, B; lane 2). Perhaps in MAFF 103036, chromosomal fragmentation resulted in relocation of *AVR2* and *AVR3* to an independent small chromosome. All race 2 and race 3 isolates carried *AVR2* or *avr2* and *AVR3* on chromosomal DNA, but none of them had the *AVR1* or *avr1*.

Mobile elements, together with point mutation in the gene [9], [24], [25], are involved in the loss-of-function of *AVR* in fungal plant pathogens such as *Magnaporthe oryzae* and *Cladosporium fulvum* [26], [27], [28], [29], [30]. Generally, mobile elements play a role in duplication and translocation of the genes/genomic regions in the genome [20], [31], sometimes they cause genetic mutations. *AVR* genes often locate on mobile element-rich regions in fungal plant pathogens, such as *M. oryzae* [32], *Leptosphaeria maculans* [24], *Blumeria graminis* [33], and *F. oxysporum* [34]. In *Phytophthora infestans*, more than five hundreds of potential avirulence genes carrying RxLR motif located in mobile element-rich genomic regions [35]. Moreover, in *FOL* NRRL 24936 (race 2), a large amount of mobile elements are located on the lineage specific (LS) chromosomes such as Chr03, Chr06, Chr14 (2.2 Mb; the small chromosome carrying *AVR2* and *AVR3*) and Chr15. Of the 72 *Hormin* elements, 37 are located on LS chromosomes of NRRL 34936 (Fig. S3).

Unlike other fungal isolates, it is easy to speculate how races emerged sequentially in *FOL* due to its simple combinations of *AVR* genes and the small number of races. Based on the arms race model [36], *FOL* and its races are considered to have emerged as follows [9] (Fig. S6): First, a nonpathogenic *F. oxysporum* isolate acquired a small chromosome carrying *AVR1*, *AVR2* and *AVR3*, and became *FOL* race 1. The deletion of the *AVR1* locus in race 1 resulted in the emergence of race 2 (– *AVR2 AVR3*), and the point mutation in *AVR2* (shown as *avr2*) in race 2 resulted in the emergence of race 3 (– *avr2 AVR3*). Refer to Table 2 for relationships among *AVR* genes, where phylogenetic groups, MAT and VCG of each isolate are also indicated. This study presented an alternative model: *AVR1* in a race 1 isolate (*AVR1 AVR2 AVR3*) lost its function by a transposon insertion, resulting in the emergence of race 2 (*avr1 AVR2 AVR3*), and race 3 (*avr1 avr2 AVR3*) emerged from the race 2 as a result of the point mutation (G121A) in *AVR2* (Fig. S6). If this scenario describes how KoChi-1 emerged, then where might it have happened? Soilborne pathogens are often carried with seed [1]. KoChi-1 may have been imported on tomato seeds from a production field because we have not found race 2 isolates carrying *AVR1* truncated by *Hormin*, so far, in Japan. There still is the possibility that KoChi-1 evolved via race 2 from a race 1 isolate belonging to the A2 clade in a particular field in Kochi Prefecture. Analysis of more isolates from Kochi, and seed production fields, will be necessary to test these hypotheses.

## Materials and Methods

### Fungal and plant materials

We sampled diseased tomato (cv. Momotaro-Fight) at a greenhouse in Hidaka, Kochi Prefecture, Japan (latitude, N33°31′53.0"; longitude, E133°21′57.3"; altitude, 32 m) on 4 Feb. 2009. Sampling was permitted by the owner of the private land and greenhouse. No other specific permits were required for the described field study. Moreover, the field study did not involve endangered or protected species. All of the isolates obtained from diseased individuals at the field were identified as *F. oxysporum* based on morphological characteristics [19]. In addition, all isolates showed identical phenotypes including virulence, mating type (MAT), vegetative compatibility (VC), combination of avirulence genes (*AVR*) and sequence of rDNA-IGS and rDNA-ITS regions. One representative isolate (KoChi-1) was chosen for this study. *FOL* race 1 (MAFF 305121, Japan), race 2 (JCM 12575, Imaichi, Tochigi, Japan, 1988) and race 3 (Chz1-A, Yatsushiro, Kumamoto, Japan, 2006) isolates were used as controls. OSU-451B (race 1, VCG 0031; a gift from H. C. Kistler, USDA and University of Minnesota, USA), MN-66 (race 2, VCG 0030+0032; a gift from H. C. Kistler) and H-1-4 (race 3, VCG 0033; a gifte from Y. Hosobuchi, Sakata Seed, Japan) were used for vegetative compatibility group (VCG) determination. OSU-451B and MN-66 were imported to Japan under special permission of Ministry of Agriculture, and Forestry and Fisheries of Japan. All isolates were stored in 25% glycerol at −150°C.

Four race differential cultivars of tomato; Ponderosa (Noguchi Seed, Saitama, Japan), Momotaro (Takii seeds, Kyoto, Japan), Walter (gifted from National Institute of Vegetable and Tea Science, Mie, Japan) and Block (Sakata Seed, Yokohama, Japan) were used. Ponderosa (*i i2 i3*) is susceptible to all *FOL* races, Momotaro (*I i2 i3*) is resistant to *FOL* race 1 but susceptible to races 2 and 3, Walter (*I I2 i3*) is resistant to races 1 and 2 but susceptible to race 3, and Block (*I I2 I3*) is resistant to all races.

### Pathogenicity assay

Race differential tomato cultivars were used to evaluate *FOL* pathogenicity. Each isolate was cultured on potato sucrose broth (PSB) for 5 days at 25°C and 120 rpm, and conidial suspensions (1.0×10^7^ conidia/ml) were prepared. Two seeds of each cultivar were sown to soil (Kureha Soil, Kureha, Iwaki, Japan) in a plastic pot (7 cm-diam.) and were maintained in a growth chamber (16 hours light at 28°C/8 hours dark at 25°C). Roots of 15-day-old tomato were injured, dipped in a conidial suspension for 5 min, and replanted to well-moistened soil. Two weeks later, external symptoms of each plant were evaluated as follows: 0, no wilt or yellowing; 1, lower leaves yellowing; 2, lower and upper leaves yellowing; 3, lower leaves yellowing and wilting and upper leaves yellowing; 4, all leaves wilted and yellowing or dead.

### DNA extraction and standard PCR

Fungal genomic DNA (gDNA) was extracted using the protocol described earlier [37], [38] with modifications.

Fragments of rDNA-ITS (521 bp) and IGS (598 bp) regions were amplified using primer sets ITS1/ITS4 [39] and FIGS11/FIGS12 [16], respectively (Table 3). We also amplified fragments of ca. 800 bp of *AVR1*, ca. 300 bp of *AVR2* and ca. 900 bp of *AVR3* using primer sets SIX4F/SIX4R, SIX3-F1/SIX3-R2 and P12-F2/P12-R1, respectively (Table 3). Each reaction mixture of 20 µl contained 20 ng of gDNA, 2.0 µl of 10×buffer (Takara Bio, Ohtsu, Japan), 1.6 µl of 2.5 mM (each) dNTPs (Takara-Bio), 8 pM of each primer, and 0.5 U of *Ex-Taq* polymerase (Takara Bio). Thermal conditions were as follows: One incubation at 94°C for 2 min; 30 cycles of: denaturation at 94°C for 30 s, annealing at 60°C for 30 s, and elongation at 72°C for 30 s; and a final extension at 72°C for 7 min. To amplify the fragment (ca. 2.0 kb) of the *AVR1* locus by SIX4f-F2/SIX4f-R2 (Table 3), we modified the annealing temperature and extension time to 45°C and 2 min, respectively.

### Sequencing

PCR amplicons purified with EXOSAP-IT (USB, Cleveland, USA) or 100 ng of plasmids were subjected to sequencing reaction using BigDye® Terminator v3.1 Cycle Sequencing Kit (Applied Biosystems) and analyzed with a 3130×l Genetic Analyzer (Applied Biosystems). Sequence was arranged with GENETYX ver. 13 (Genetyx, Tokyo, Japan).

### Phylogenetic analysis

Nucleotide sequences of the rDNA-IGS fragment from KoChi-1 were aligned with those from other *FOL* isolates using CLUSTAL X 2.0 [40]. We constructed the phylogeny by the neighbor joining (NJ) method [41] based on Kimura's two-parameter model [42], using MEGA v. 4 [43]. The statistical reliability of each node was assessed using 1000 bootstrap iterations. *F. sacchari* (synonym, *Gibberella sacchari*; mating population B of the *G. fujikuroi*-species complex) FGSC 7610 was used as an outgroup. All sequence data except for KoChi-1 were cited from the NCBI database.

### Mating type (MAT) and vegetative compatibility group (VCG) determination

Mating type, MAT1-1 or MAT1-2, was determined by PCR using Gfmat1a/Gfmat1b or GfHMG11/GfHMG12, respectively (Table 3). The reaction mixture was prepared as described in the section of Standard PCR, reaction conditions were set as follows: One incubation at 94°C for 2 min; 30 cycles of: denaturation at 94°C for 30 s, annealing at 58°C for 30 s, and elongation at 72°C for 45 s; and a final extension at 72°C for 6 min.

We also identified the VCG type of each isolate. To date, four vegetative compatibility groups (VCGs), 0030+0032, 0031, 0033 and 0035, have been reported in *FOL* [5]. The complementation test was performed using the tester isolates, OSU-451B (VCG 0031), MN-66 (VCG 0030+0032) and H-1-4 (VCG 0033). Each nitrate nonutilizing (*nit*) mutant of each isolate (*nit1* and NitM) was prepared, and a compatibility test was performed following the procedures described previously [44].

### Gene expression analysis

Tomato cv. Ponderosa was inoculated with *F. oxysporum* as described in the section entitled “Pathogenicity assay”. Eight days after inoculation, we vigorously washed the tomato roots with sterilize water. After drying with paper towels, roots were crushed in liquid nitrogen and total RNA was extracted with the SV Total RNA Isolation System (Promega) following the manufacturer's manual. From the extracted total RNA, cDNA was synthesized using TaKaRa RNA PCR Kit (AMV) Ver. 3.0 (TaKaRa Bio). Expression of target genes was examined with 5 ng of cDNA. To investigate expression of *AVR1*, *avr1*, *AVR2*, *AVR3*, *FEM1* and the tomato actin gene, primer sets SIX4F/SIX4R, SIX4F/hornet-like2, FP962/FP963, and FP157/FP158, and Actin-f/Actin-r (Table 3) were used for PCR, respectively. *FEM1* [45], [46] and Actin [47] were used as controls for fungal and plant genes, respectively. Negative controls substituted sterile water for conidial suspension. Reaction mixtures were prepared as described above. Thermal conditions were: One incubation at 94°C for 2 min; 35 cycles of: denaturation at 94°C for 30 s, annealing at 57°C for 30 s, and elongation at 72°C for 30 s; and a final extension at 72°C for 7 min.

### Complementation with *AVR1* using Agrobacterium tumefaciens-mediated transformation (ATMT)

The *AVR1* gene of *FOL* race 1 Fol004 was integrated into the KoChi-1 genome ectopically by the ATMT method. Transformation using the binary vector pPHSIX4c (carrying about 2.0 kb of *AVR1* locus and phleomycin resistance gene) [8] was carried out following the procedure described earlier [10] with minor modifications. To suppress the growth of *Agrobacterium* after transformation, we used 25 µg/ml Melopen (Dainippon Sumitomo Phama, Osaka, Japan) and 50 µg/ml Zeocin (Invitrogen, San Diego, USA), respectively.

### Contour-clamped homogeneous electric field (CHEF)-gel analysis

In addition to KoChi-1, we used several race 1 isolates; MAFF 305121 (Japan), MAFF 103036 (Japan), 73 (Italy; gift from Corby H. Kistler) and Ita3 (Italy; gift from Giorgia Ferro, The Regional Center For Agricultural Experimentation and Assistance, Italy): race 2 isolates; NRRL 24936 (Spain; gift from A. Di. Pietro, University of Cordoba, Spain) and JCM 12575 (Japan) and race 3 isolate, Chz1-A (Japan). Protoplasts were prepared following [48] with slight modification; we used enzyme solution containing 1.0% Lysing enzymes (Sigma, St. Louis, USA) and 1.0% Driselase (ASKA Pharmaceutical, Tokyo, Japan) for digestion of fungal cell wall, and Proteinase K (Nakarai Tesk, Kyoto, Japan) was used for plug purification.

CHEF gel electrophoresis was performed in 1.0% Sea Kem gold agarose gel (FMC BioProducts, Rockland, USA) with CHEF Mapper® XA Pulsed Field Electrophoresis System (BioRad, Hercules, USA). The condition to separate chromosomes was as described earlier [34] with slight modification; 260 hours run at 8°C, 1200–4800 s switch time at 1.5 V/cm. The running buffer 0.5xTBE was refreshed every 2 days. Chromosomes of *Schizosaccharomyces pombe* (BioRad) and *Saccharomyces cereviciae* (BioRad) were used as DNA size markers. The gel was stained with ethidium bromide to visualize chromosomes after running electrophoresis.

### Southern blot analysis

Probes for *AVR1*/*avr1*, *AVR2*/*avr2* and *AVR3* were prepared using SIX4F/SIX4R, SIX3-F1/SIX3-R2 and P12-F2/P12-R1, respectively. For genomic Southern hybridization, 8.0 µg gDNA were digested with *Nde*I and *Bss*HII, and incubated overnight at 37°C. The following procedure was performed as described earlier [49], note that Whatman Nytran SuPerCharge (SPC) nylon blotting membranes (Sigma) was used in this study.

The CHEF-gel was treated with 0.25 N HCl for 30 min, followed by denaturation buffer (0.5 M NaOH. 1.5 M NaCl), and the digested chromosomes were transferred to a nylon membrane (Hybond N+; Amersham, Amersham, UK) washed in 0.4 M NaOH for about 72 hours. The following procedure after transfer was performed as early study [49]. For stripping the hybridized probe, the used membrane was washed twice, for 15 min each, with 0.2 M NaOH, 0.1% SDS at 37°C, then the membranes were soaked in 2×SSC for 5 min and dried.

## Supporting Information

Figure S1 **Fusarium wilt of tomato caused by** ***F*** **.** ***oxysporum*** **f. sp.** ***lycopersici*** **in Kochi, Japan.** (A) Location of the wilt disease emerged. Asterisk at the tip of bar presents Hidaka, Kochi Prefecture, Japan (latitude, N33°31′53.0"; longitude, E133°21′57.3"; altitude, 32 m). (B) Diseased tomato cultivar Momotaro-Fight (*I I2 i3*) in a greenhouse in Hidaka, Kochi prefecture, Japan. The diseased tomato plants wilted and the color of the leaves turned yellow. Severely diseased plants did not survive and white hyphae were observed on the lower part of their stems.
(TIF)

Figure S2 **Phylogenetic relationship of tomato wilt fungus (** ***FOL*** **) isolates in Japan.** KoChi-1 and other *FOL* races 1∼3 isolates obtained in Japan were used. Race, the source, mating type (MAT) and vegetative compatibility group (VCG) were described in parentheses at the end of the isolates name. A hyphen indicates incompatible isolates with VCG testers. *Gibberella fujikuroi* strain FGSC 7610 was used as the outgroup. The phylogeny was constructed based on Kimura's two-parameter [42] as nucleotide substitution model using MEGA v. 4 [43]. Bootstrap iterations are 1000 replications, the values are indicated at tree nodes. Bootstrap values greater than 70% are shown beside nodes. The *FOL* clades A1, A2 and A3 are consistent with the previous study [16]. All sequence data are in the DDBJ/EMBL/GenBank databases; KoChi-1 (AB674508), MAFF 103043 (AB106032), JCM 12575 (AB106027), SUF 1330 (AB106035), MAFF 103038 (AB106031), MAFF 305121 (AB106021), MAFF 103036 (AB106020), MAFF 727501 (AB106022), Chz1-A (AB373819), F-1-1 (AB106037) and FGSC 7610 (AB106061).
(TIF)

Figure S3 **Southern blot analysis to detect** ***AVR1*** **and** ***avr1*** **genes of KoChi-1 transformants.** The probe was prepared using a primer set SIX4F/SIX4R (Table 3, Fig. 2C), each 8.0 µg gDNA was digested with *Nde*I. Race 1, MAFF 305121 (*AVR1 AVR2 AVR3*); race 3, KoChi-1 (*avr1 avr2 AVR3*); transformants, K-B-b, K-2-11 and K-2-12 (*avr1 AVR1 avr2 AVR3*).
(TIF)

Figure S4 **The deduced amino acid sequences of** ***AVR1*** **in race 1 and** ***avr1*** **in KoChi-1.** The AVR1 is composed of 242 amino acids. The deduced amino acid sequence of *AVR1* with *Hormin* in KoChi-1 revealed a chimeric AVR1 composed of 175 amino acids (avr1) that may not function as AVR1. Black and orange characters show the amino acids encoded by *AVR1* and *Hormin*, respectively. Asterisks show the homologous amino acid.
(TIF)

Figure S5 ***Hormin*** **distributes on every chromosome of** ***FOL*** **race 2 NRRL 34936.** Red arrowheads show the location of *Hormin*. The figures of the *FOL* chromosome was cited from the website of Broad Institute (http://www.broadinstitute.org/annotation/genome/fusarium_group/MultiHome.html).
(TIF)

Figure S6 **A novel path of emergence of** ***FOL*** **races proposed in this study.**
(TIF)
