# Supplementary figures and images for: A Genetic Mechanism for Emergence of Races in Fusarium oxysporum f. sp. lycopersici: Inactivation of Avirulence Gene AVR1 by Transposon Insertion
Source: PLoS One. 2012 Aug 27;7(8):e44101. doi: 10.1371/journal.pone.0044101 (PMC3428301; doi:10.1371/journal.pone.0044101)

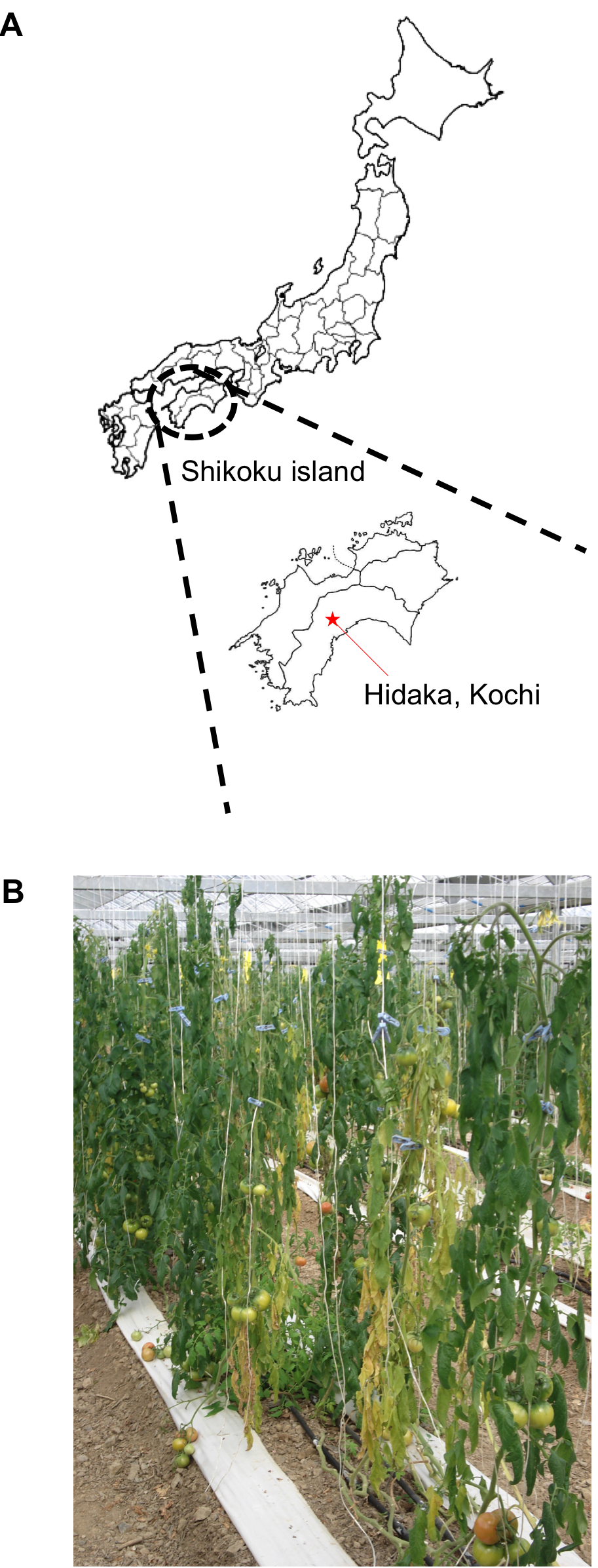

Supplement: Figure S1 — Fusarium wilt of tomato caused by F . oxysporum f. sp. lycopersici in Kochi, Japan. (A) Location of the wilt disease emerged. Asterisk at the tip of bar presents Hidaka, Kochi Prefecture, Japan (latitude, N33°31′53.0"; longitude, E133°21′57.3"; altitude, 32 m). (B) Diseased tomato cultivar Momotaro-Fight (I I2 i3) in a greenhouse in Hidaka, Kochi prefecture, Japan. The diseased tomato plants wilted and the color of the leaves turned yellow. Severely diseased plants did not survive and white hyphae were observed on the lower part of their stems. (TIF) [file pone.0044101.s001.tif]

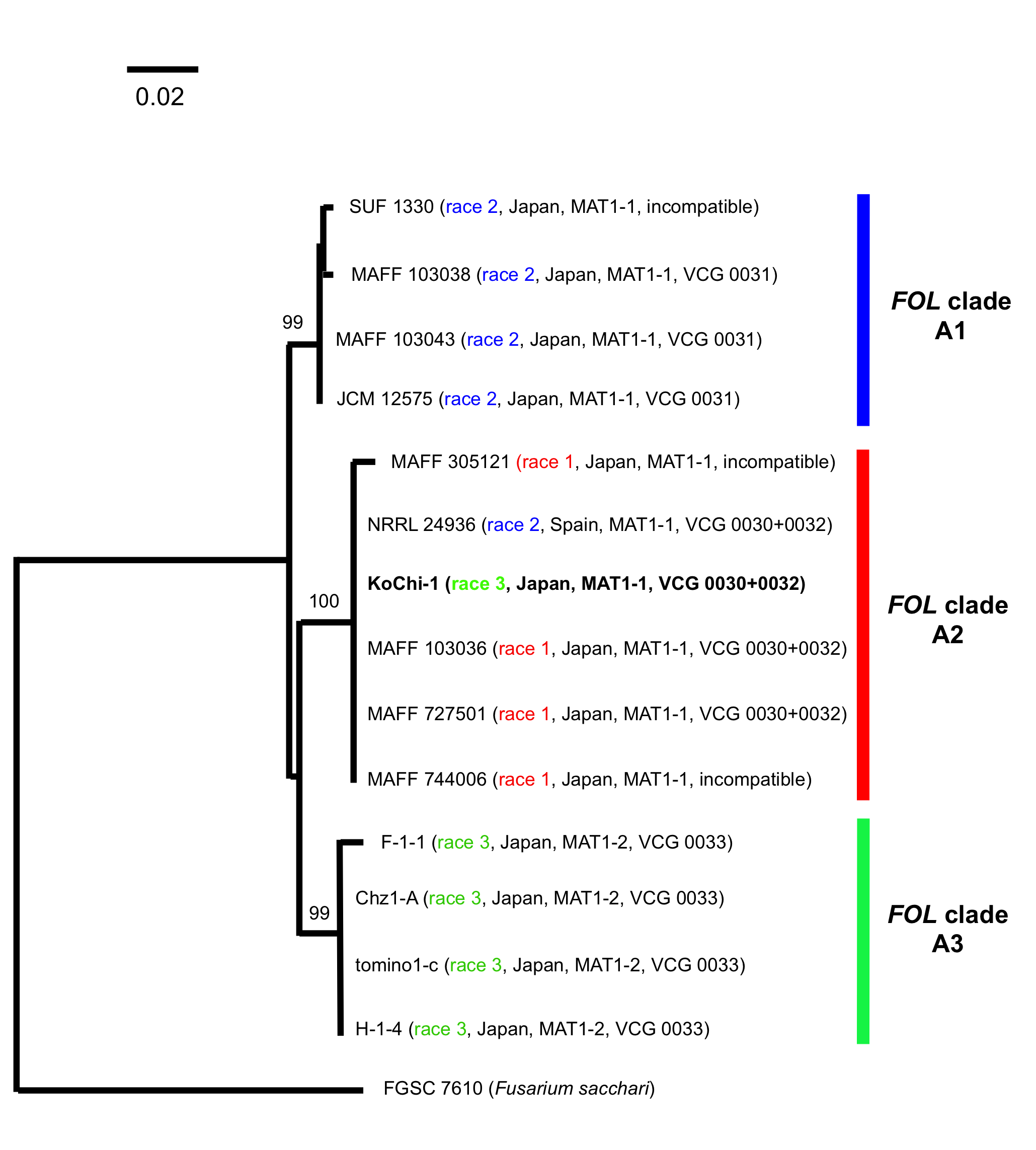

Supplement: Figure S2 — Phylogenetic relationship of tomato wilt fungus ( FOL ) isolates in Japan. KoChi-1 and other FOL races 1∼3 isolates obtained in Japan were used. Race, the source, mating type (MAT) and vegetative compatibility group (VCG) were described in parentheses at the end of the isolates name. A hyphen indicates incompatible isolates with VCG testers. Gibberella fujikuroi strain FGSC 7610 was used as the outgroup. The phylogeny was constructed based on Kimura's two-parameter [42] as nucleotide substitution model using MEGA v. 4 [43]. Bootstrap iterations are 1000 replications, the values are indicated at tree nodes. Bootstrap values greater than 70% are shown beside nodes. The FOL clades A1, A2 and A3 are consistent with the previous study [16]. All sequence data are in the DDBJ/EMBL/GenBank databases; KoChi-1 (AB674508), MAFF 103043 (AB106032), JCM 12575 (AB106027), SUF 1330 (AB106035), MAFF 103038 (AB106031), MAFF 305121 (AB106021), MAFF 103036 (AB106020), MAFF 727501 (AB106022), Chz1-A (AB373819), F-1-1 (AB106037) and FGSC 7610 (AB106061). (TIF) [file pone.0044101.s002.tif]

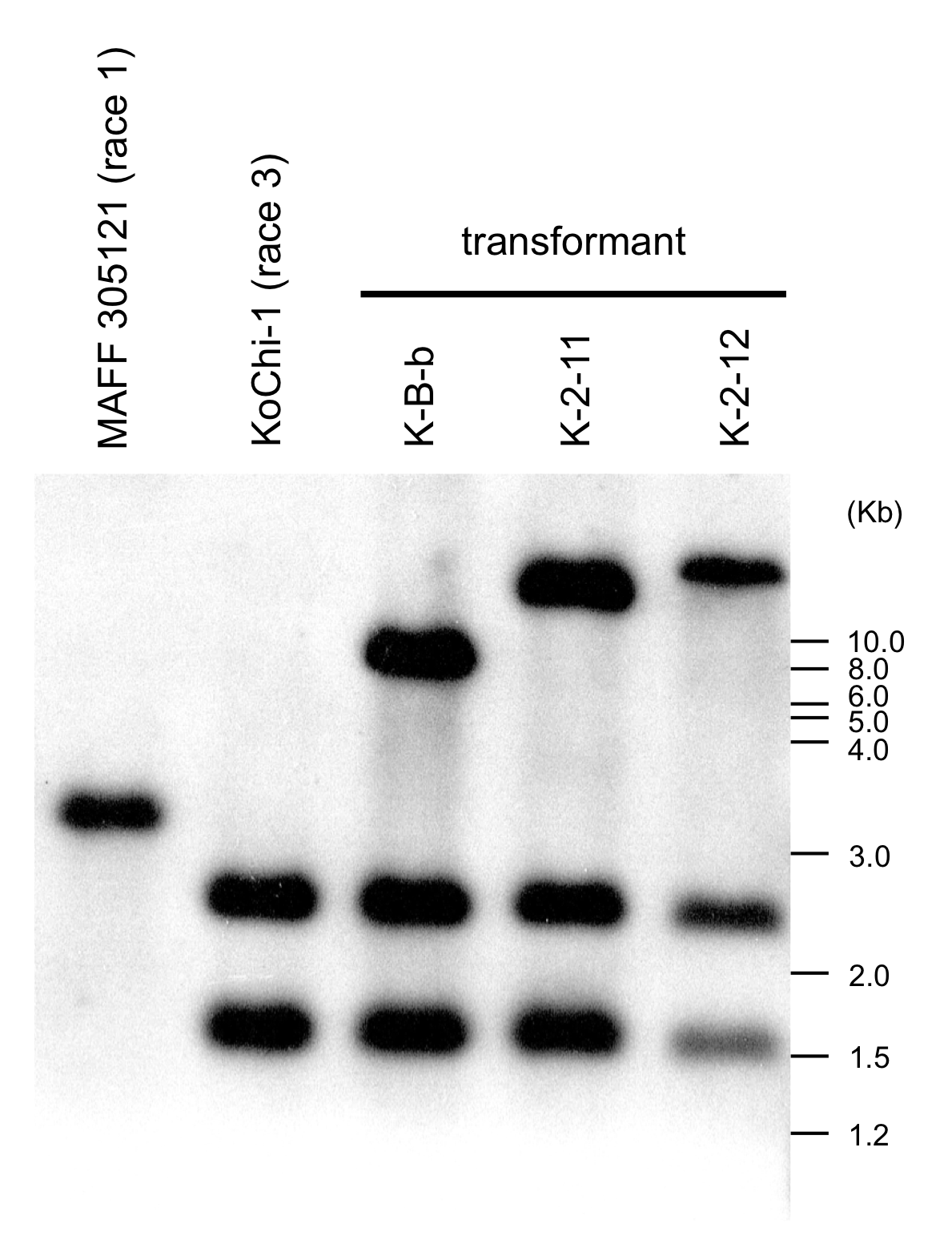

Supplement: Figure S3 — Southern blot analysis to detect AVR1 and avr1 genes of KoChi-1 transformants. The probe was prepared using a primer set SIX4F/SIX4R (Table 3, Fig. 2C), each 8.0 µg gDNA was digested with NdeI. Race 1, MAFF 305121 (AVR1 AVR2 AVR3); race 3, KoChi-1 (avr1 avr2 AVR3); transformants, K-B-b, K-2-11 and K-2-12 (avr1 AVR1 avr2 AVR3). (TIF) [file pone.0044101.s003.tif]

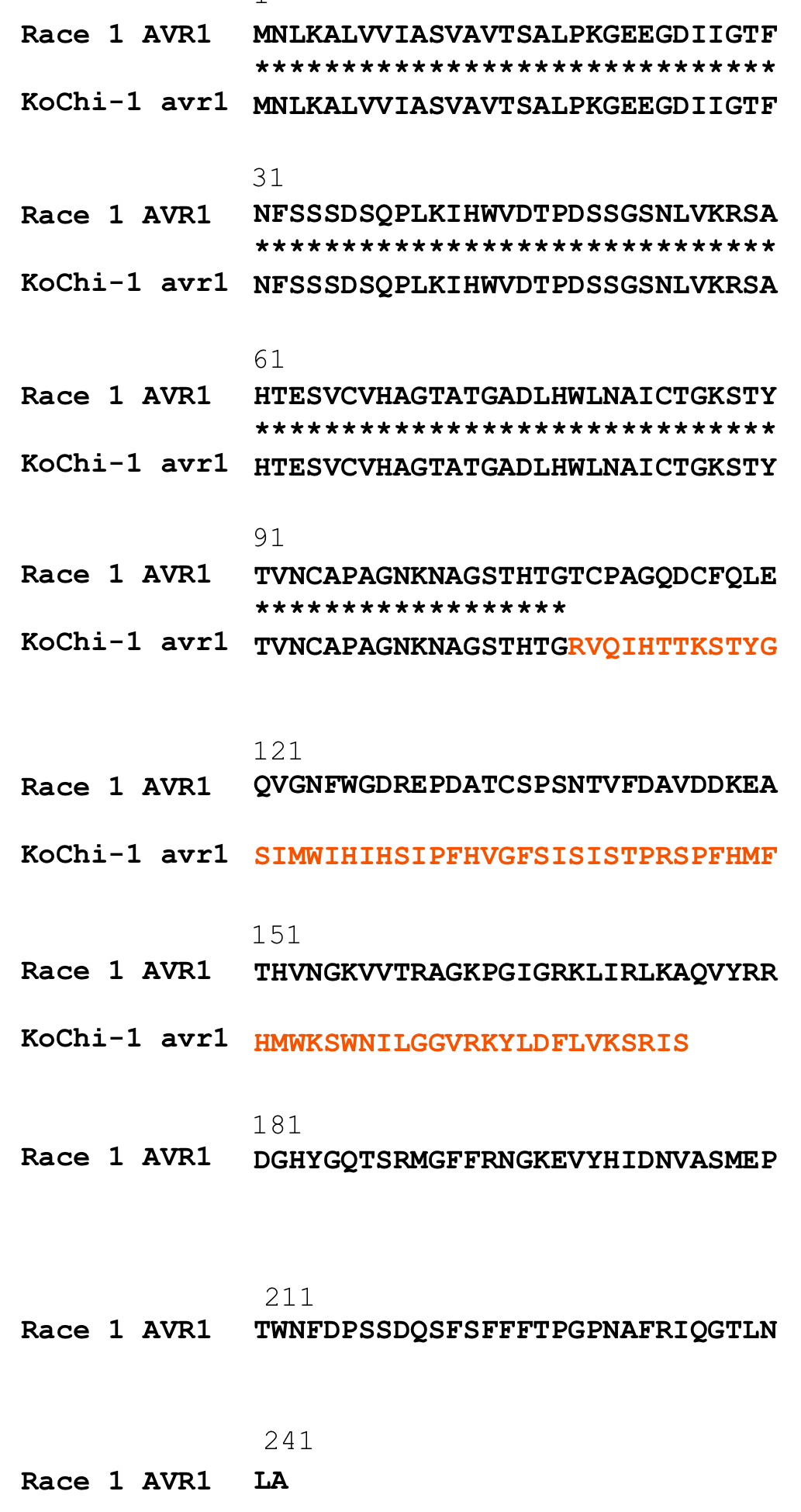

Supplement: Figure S4 — The deduced amino acid sequences of AVR1 in race 1 and avr1 in KoChi-1. The AVR1 is composed of 242 amino acids. The deduced amino acid sequence of AVR1 with Hormin in KoChi-1 revealed a chimeric AVR1 composed of 175 amino acids (avr1) that may not function as AVR1. Black and orange characters show the amino acids encoded by AVR1 and Hormin, respectively. Asterisks show the homologous amino acid. (TIF) [file pone.0044101.s004.tif]

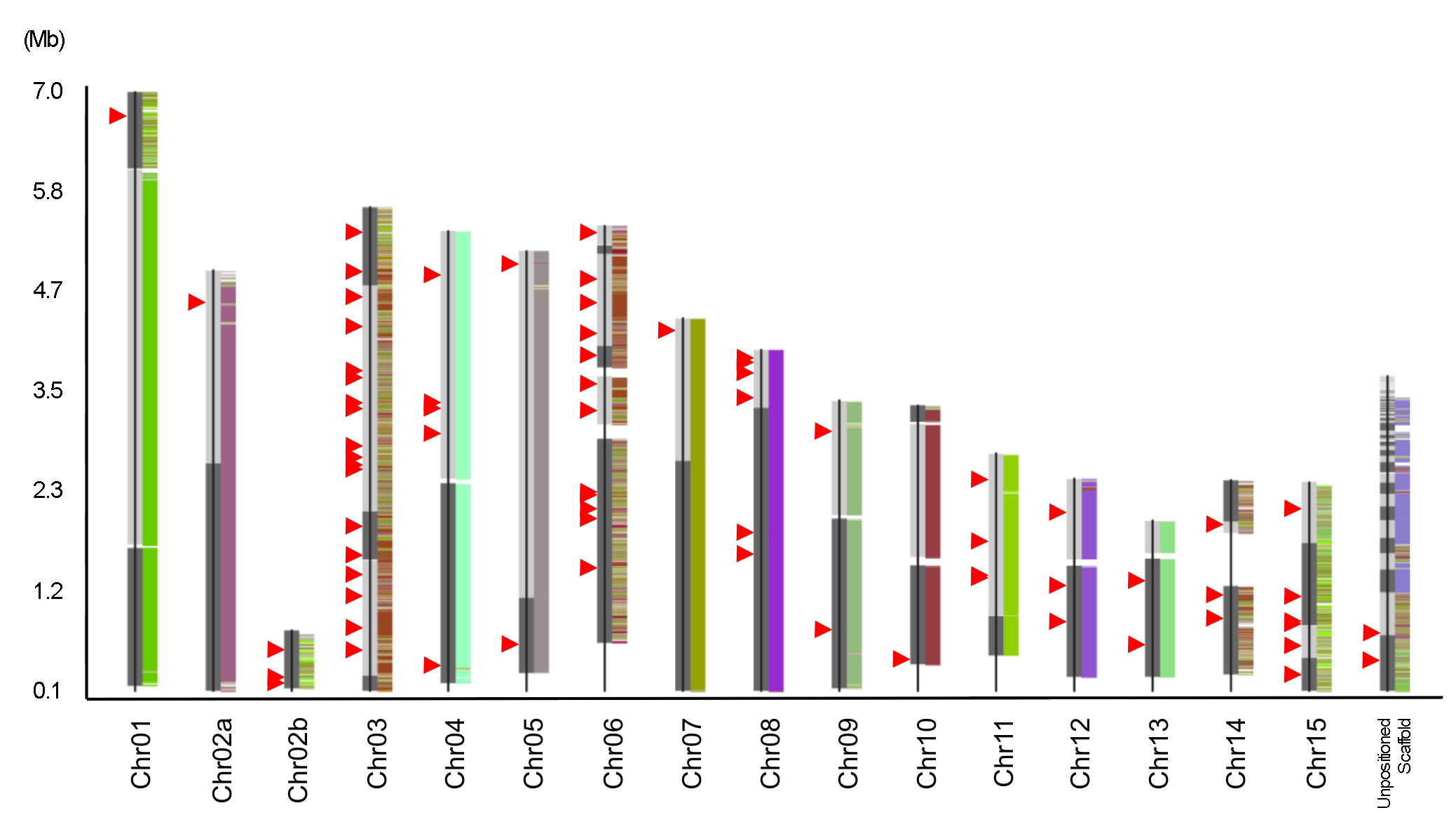

Supplement: Figure S5 — Hormin distributes on every chromosome of FOL race 2 NRRL 34936. Red arrowheads show the location of Hormin. The figures of the FOL chromosome was cited from the website of Broad Institute (http://www.broadinstitute.org/annotation/genome/fusarium_group/MultiHome.html). (TIF) [file pone.0044101.s005.tif]

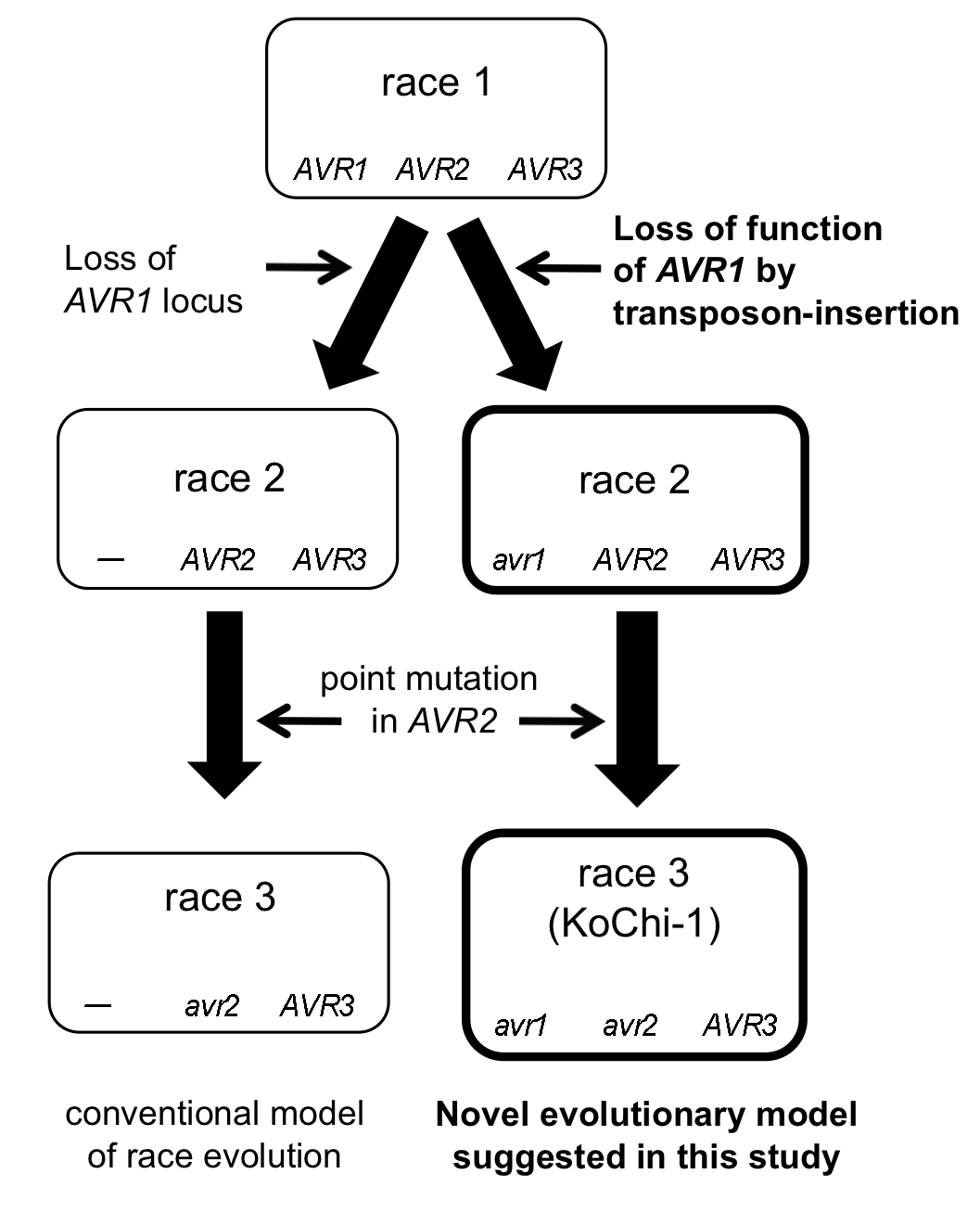

Supplement: Figure S6 — A novel path of emergence of FOL races proposed in this study. (TIF) [file pone.0044101.s006.tif]
